# Epicardial adipose tissue as a determinant of heart failure prognosis: insights across ejection fraction phenotypes

**DOI:** 10.1186/s12933-026-03126-y

**Published:** 2026-03-09

**Authors:** Maksymilian Nowakowski, Maciej Mazuruk, Łukasz Nogajski, Maciej Mączewski, Hanna Czerwińska, Mikołaj Kurpias, Michał Mączewski, Aleksandra Paterek

**Affiliations:** 1 Department of Clinical Physiology, Centre of Postgraduate Medical Education, Warsaw, Poland; 2 Student’s Cardiovascular Scientific Club “Kardioplegia”, Medical University of Warsaw, Warsaw, Poland; 3 Heart Failure and Transplantology Department, Mechanical Circulatory Support and Transplant Department, National Institute of Cardiology, Warsaw, Poland

**Keywords:** Epicardial adipose tissue, Heart failure, Imaging, Ventricular arrhythmias

## Abstract

Epicardial adipose tissue (EAT) is a metabolically active fat depot located between the myocardium and visceral pericardium, directly interacting with cardiomyocytes and coronary vasculature. Emerging evidence suggests that EAT plays a significant role in the pathophysiology and prognosis of heart failure (HF) across different left ventricular ejection fraction (LVEF) phenotypes. This review summarizes current data on the prognostic role of EAT in HF, including volume, thickness, and density, measured by echocardiography, computed tomography, and cardiac magnetic resonance imaging. In HF with preserved (HFpEF) and mildly reduced ejection fraction (HFmrEF), increased EAT consistently associates with adverse outcomes, including higher rates of hospitalization and mortality, independent of systemic obesity. In HF with reduced ejection fraction (HFrEF), the relationship is more complex, with studies showing both protective and detrimental associations depending on EAT quantity, density, and spatial distribution. EAT also appears to contribute to ventricular arrhythmogenesis, particularly in patients with preserved myocardial structure. Mechanistically, EAT may promote inflammation, fibrosis, and electrophysiological remodeling, while moderate amount EAT may exert metabolic or mechanical protection. Overall, EAT emerges as a promising imaging-derived biomarker for risk stratification in HF, highlighting the need for phenotype-specific evaluation and potential therapeutic targeting. Future studies should focus on EAT quality, remodeling, and its interaction with myocardial tissue to guide individualized HF management.

**Graphical abstract:**

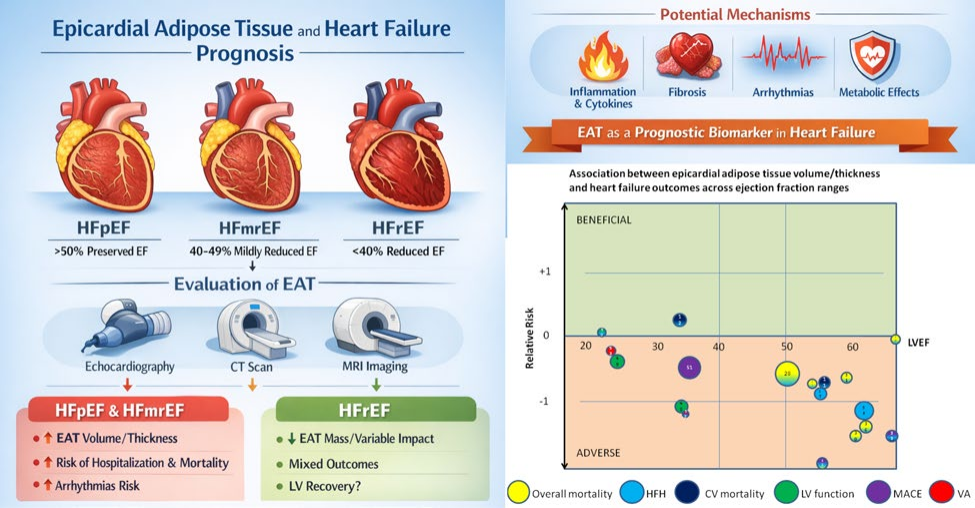

## Heart failure: characterization of different subtypes

Heart failure (HF) is a clinical syndrome characterized by typical symptoms (e.g., dyspnea, fatigue, peripheral edema) and signs resulting from elevated intracardiac pressures and/or inadequate cardiac output) [1]. HF is commonly categorized into heart failure with reduced ejection fraction (HFrEF) and heart failure with preserved ejection fraction (HFpEF), with an intermediate phenotype—heart failure with mildly reduced ejection fraction (HFmrEF)—falling between these two entities [2].

**Fig. 1:**
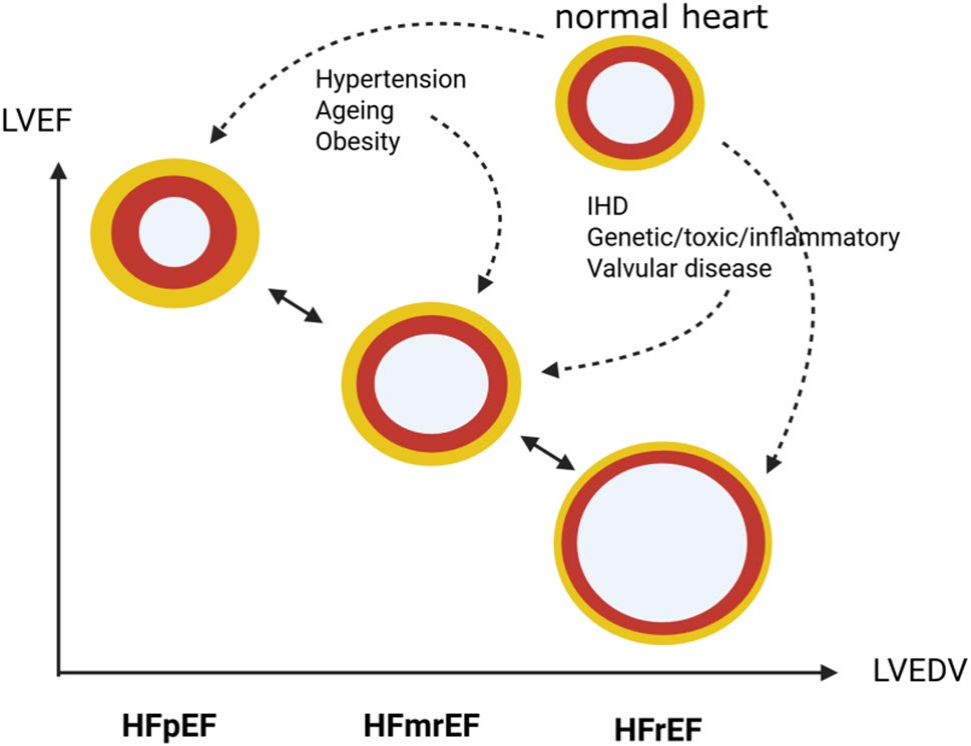
Heart failure phenotypes Heart failure is commonly categorized based on LVEF, into heart failure with preserved ejection fraction (HFpEF), LVEF > 50%), heart failure with mildly reduced ejection fraction (HFmrEF, LVEF 40–49) and heart failure with reduced ejection fraction (HFrEF, LVEF < 40%). HFpEF is characterized by preserved EF, LV hypertrophy, diastolic dysfunction, while HFrEF by impaired EF, LV dilation, often LV wall thinning. Epicardial adipose tissue (a yellow layer covering the red LV myocardium) is increased in HFpEF and often reduced in HFrEF. HFmrEF is a transitional phenotype that shares features of both HFrEF and HFpEF, representing worsening HFpEF or improving HFrEF. IHD, ischemic heart disease; LVEDV, left ventricular end-diastolic volume; LVEF, left ventricular ejection fraction. The left ventricular wall is shown as a red circle, enclosing the lumen, with a surrounding yellow layer of epicardial adipose tissue

HFrEF (left ventricular ejection fraction [LVEF] ≤ 40%) is primarily driven by impaired left ventricular systolic function due to eccentric ventricular remodeling and reduced contractility, most often resulting from ischemic heart disease or dilated cardiomyopathy (Fig. 1). Its pathophysiology is dominated by neurohormonal activation, leading to progressive ventricular dilation and reduced stroke volume [1].

HFpEF (LVEF ≥ 50%) is characterized by impaired ventricular relaxation and increased myocardial stiffness despite preserved or near-normal systolic function. Its pathophysiology involves concentric remodeling, coronary microvascular dysfunction, systemic inflammation, and abnormal ventricular–vascular coupling, and is commonly associated with aging, hypertension, obesity, and diabetes mellitus [3].

HFmrEF (LVEF 41–49%) represents an intermediate phenotype with overlapping features of systolic and diastolic dysfunction. Patients with HFmrEF often share clinical characteristics, comorbidity profiles, and treatment responsiveness more closely resembling HFrEF than HFpEF, suggesting partial reversibility of systolic impairment in a substantial proportion of patients [4].

Across all HF phenotypes, elevated cardiac filling pressures and congestion constitute the central hemodynamic abnormalities responsible for symptoms. However, patterns of structural remodeling differ, with eccentric dilation predominating in HFrEF, concentric hypertrophy in HFpEF, and mixed or mild remodeling in HFmrEF. Thus, while all forms of HF share common clinical manifestations, they differ substantially in underlying myocardial mechanics, dominant pathophysiologic mechanisms, and response to guideline-directed therapies [1].

## Heart failure outcomes

**Fig. 2:**
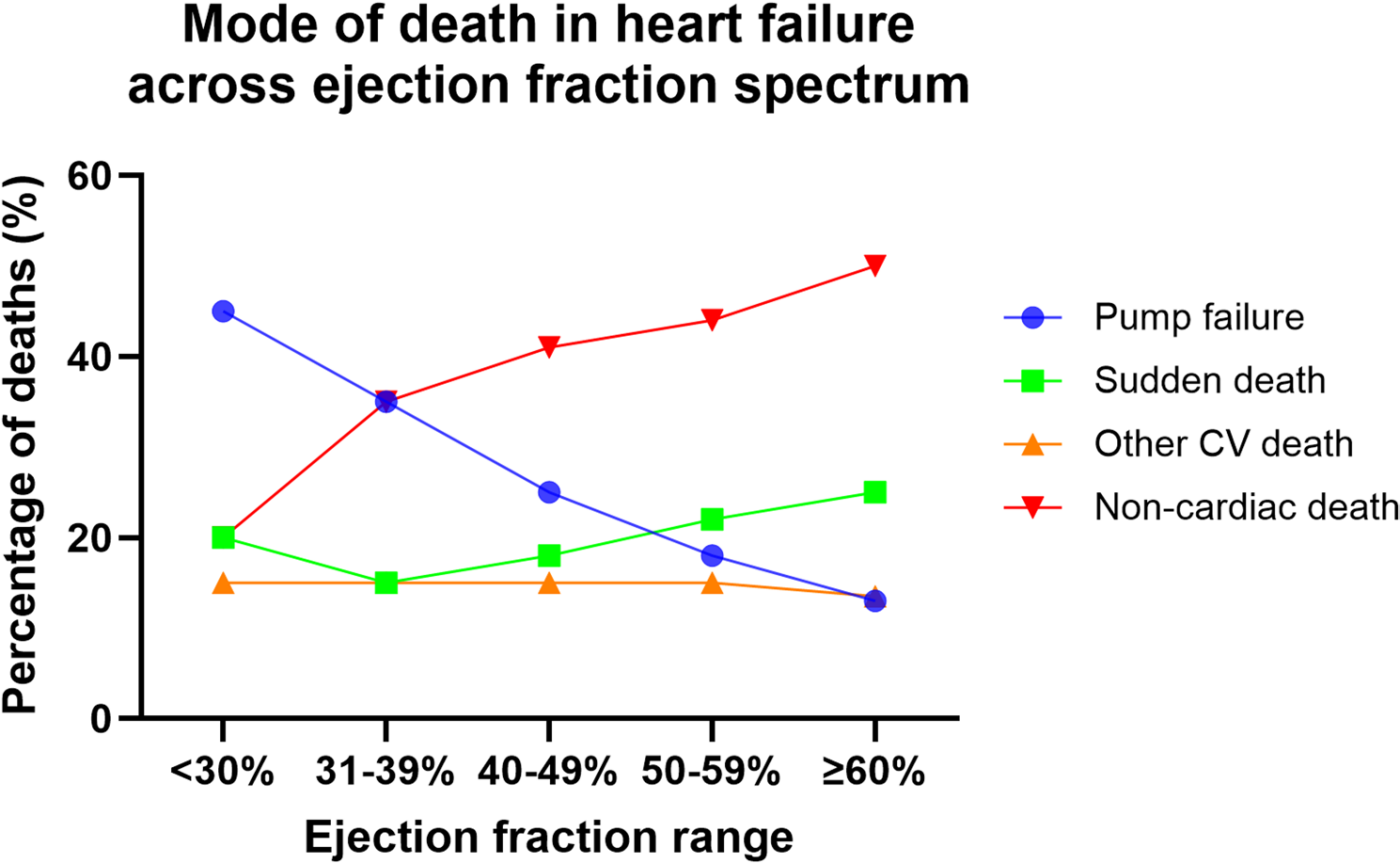
Mode of death in heart failure depending on specific ejection fraction range pump failure deaths dominate at low left ventricular ejection fraction (LVEF) and decrease progressively as LVEF increases. Sudden death shows a U-shaped / rising pattern toward higher LVEF ranges, consistent with heart failure with preserved ejection fraction (HFpEF) trial observations. Non-cardiac death steadily increases with higher LVEF and dominates at EF ≥ 50–60%. Other cardiovascular (CV) deaths remain relatively stable across LVEF strata. A schematic overview based on findings from multiple clinical trials and registry data [6–9]

Despite major therapeutic advances HF continues to be associated with substantial mortality (Fig. 2), morbidity, and healthcare utilization across all LVEF phenotypes. Overall survival remains limited, with approximately 50% of patients dying within five years of diagnosis, although prognosis varies by LVEF [5].

HFrEF carries the highest risk of cardiovascular mortality, driven primarily by progressive pump failure and sudden cardiac death (SCD), the latter presumably of arrhythmic origin (Fig. 2) [6]. HFpEF is associated with mortality rates comparable to HFrEF but with a greater contribution from non-cardiovascular causes and comorbidities. Recurrent hospitalizations for congestion are particularly frequent in HFpEF, reflecting limited disease-modifying treatment options.

In randomized controlled trials, majority of deaths in HFpEF patients were cardiovascular (~ 60–70% of total deaths), whereas 20–30% were due to non-cardiac causes. Observational epidemiological studies, however, report lower proportions of cardiovascular deaths (∼50–60%) in HFpEF patients when compared with randomized controlled trials.

Unlike HFrEF where most of the cases of SCD are due to malignant ventricular arrhythmias (ventricular tachycardia and ventricular fibrillation), the proportion of HFpEF patients with SCD due to malignant arrhythmias remains unknown but is probably high. The mechanisms driving fatal events in HFpEF, especially SCD, remain elusive [10].

HFmrEF demonstrates intermediate outcomes in terms of mortality and hospitalization. Importantly, patients with HFmrEF often derive benefits from neurohormonal therapies similar to those seen in HFrEF, suggesting that its clinical trajectory may be more dynamic and amenable to therapeutic intervention.

Across all HF phenotypes, quality of life is markedly impaired, with exercise intolerance and symptom burden remaining prominent, especially in HFpEF. Thus, while outcomes differ by LVEF in causes of death and therapeutic responsiveness, HF remains uniformly associated with high mortality, symptom burden and healthcare resource use.

## Epicardial adipose tissue

Epicardial adipose tissue (EAT) is a fat depot located between the myocardium and the visceral layer of the pericardium, in direct contact with cardiomyocytes and encompasing large epicardial coronary vasculature (Fig. 1) [11]. This contiguity allows bidirectional paracrine and vasocrine signaling between EAT and the myocardium, a feature unique among fat depots.

EAT covers approximately 80% of the heart’s surface, with distribution mainly along the atrioventricular and interventricular grooves, and around the right (RV) and left ventricles (LV) [12]. EAT contains adrenergic and cholinergic neurons, and produces norepinephrine, potentially modulating local sympathetic activity [13,14].

Functionally, EAT may provide mechanical protection, serve as a local energy reservoir, buffer excess fatty acids, and generate heat. It also secretes a wide range of bioactive molecules, including adipokines and cytokines, which influence cardiomyocyte and fibroblast function and are altered in cardiovascular disease. EAT quantity and secretory profile are influenced by risk factors such as obesity, diabetes, and hyperlipidemia [11].

In both healthy individuals and patients with HFpEF, metabolic factors, such as visceral obesity, BMI closely correlate with total EAT volume [15,16] indicating that EAT behaves similarly to other adipose depots and remains largely under the influence of systemic metabolic regulation. However, as we and others have recently demonstrated, this correlation is lost in HFrEF [16–18]. These findings suggest that, in the context of HFrEF, alternative mechanisms may predominate in regulating EAT biology, including systemic neurohumoral activation, inflammatory mediators, or direct local interactions with the failing myocardium [16].

Overall, EAT is a highly dynamic and functionally significant cardiac fat depot, integrating metabolic, mechanical, and neurohormonal roles in cardiac physiology and pathophysiology [19].

## Imaging methods of epicardial adipose tissue

Echocardiography, cardiac magnetic resonance imaging (cMRI) and cardiac computed tomography (cCT) are established modalities for the assessment of EAT. Table 1 presents advantages and disadvantages of each of these modalities.

**Table 1:**
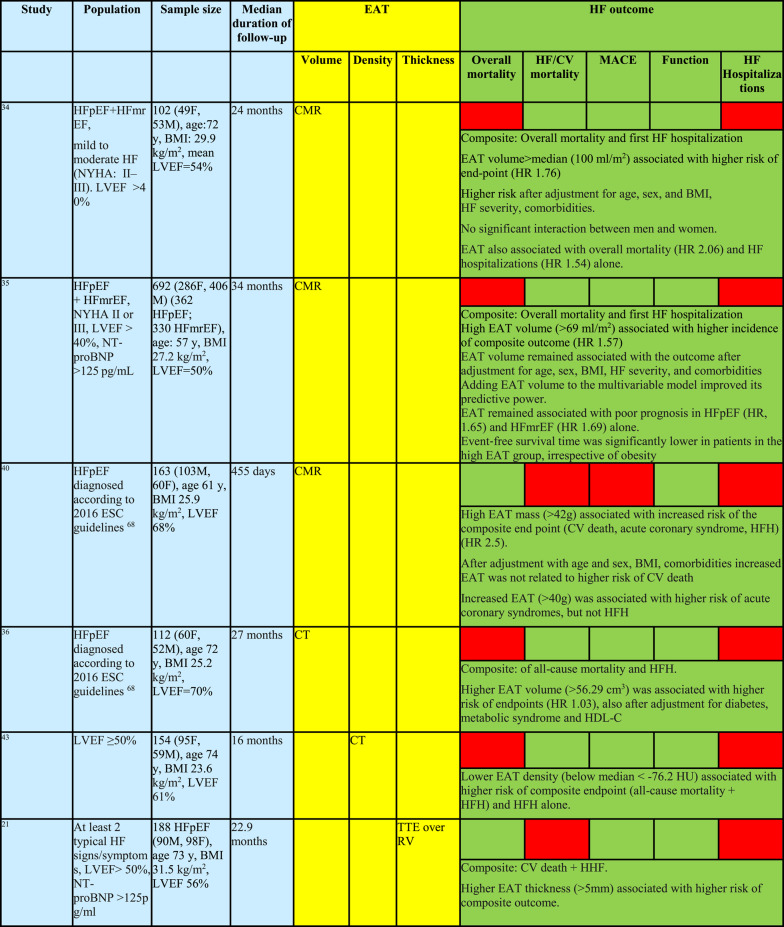

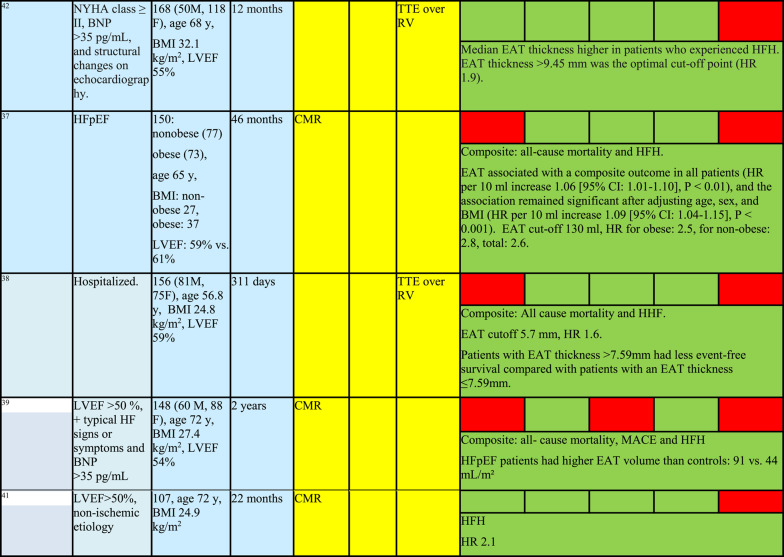

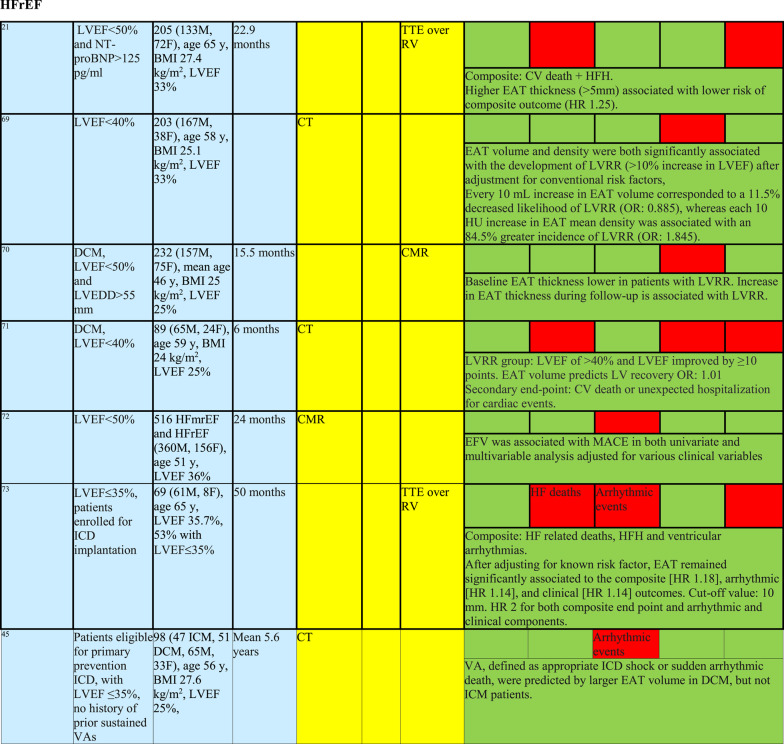
Summary of studies evaluating prognostic significance of epicardial adipose tissue across heart failure phenotypesHFpEF and HFmrEF

BMI, body mass index; CMR, cardiac magnetic resonance; CT, computed tomography; DCM, dilated cardiomyopathy, EAT, epicardial adipose tissue; F, female; HF, heart failure; HFH, heart failure hospitalizations; HFmrEF, heart failure with mildly reduced ejection fraction; HFpEF, heart failure with preserved ejection fraction; HFrEF, heart failure with reduced ejection fraction; ICD, implantable cardioverter-defibrillator; ICM, ischemic cardiomyopathy; LVEF, left ventricular ejection fraction; LVRR, left ventricular reverse remodeling; M, male; MACE, major adverse cardiovascular effects; RV, right ventricle; TTE, transthoracic echocardiography; VA, ventricular arrhythmias; y, years

Transthoracic echocardiography identifies EAT as a hypoechoic space between the outer wall of myocardium and the visceral layer of pericardium. It usually measures EAT thickness in the parasternal long axis view along the RV free wall due to its superior visibility. cMRI enables easy volumetric quantification of EAT (Table 2). Finally cCT provides excellent quantification of EAT volume and density [20].

**Table 2:** Imaging modalities to assess epicardial fat

Imaging modality	Possible measurements	Advantages	Disadvantages
cCT	Volume Thickness Area Density	Good resolution Volumetric quantification Density assessment Widely available Often performed for other indications	Ionizing radiation
cMRI	Volume Thickness Area	Good resolution Volumetric quantification No radiation or iodinated contrast required	Expense Longer scan times Contraindicated in patients with implanted devices No information on density
Echocardiography	Thickness	Widely available Extremely safe Inexpensive Often performed for other indications	No volumetric or density quantification EAT only measured over the RV Poor image quality (especially in obesity) Lower reproducibility

## Epicardial adipose tissue in heart failure

Multiple studies demonstrate that the EAT thickness over RV is reduced in patients with HFrEF compared with healthy controls [21–23], and is even more markedly reduced compared with patients with patients with HFpEF [21, 22, 24]. EAT volume or mass assessed by CMR or CT, has been reported as reduced [17, 25–28], unchanged [18] or increased [24, 29] compared to healthy subjects. However, given the markedly increased cardiac mass in HFrEF, normalization of EAT mass to myocardial mass provides a more appropriate measure of EAT accumulation. When indexed in this manner, EAT mass is consistently reduced across all studies [17, 18, 24–29]. Autopsy studies further demonstrate reduced cardiac EAT coverage in HFrEF [30], a finding we have recently confirmed using CT imaging [16].

 In contrast, EAT thickness over the RV is increased in HFpEF patients compared with healthy subjects [21, 22, 31, 32] and with HFrEF patients (see above). Consistently, both absolute and indexed EAT volume or mass are increased in HFpEF [24, 29, 33]. Unfortunately, no specific data are available for HFmrEF.

This discrepancy in EAT volume between HFpEF and HFrEF likely reflects the distinct pathophysiological profiles of these heart failure subtypes: an obesity-driven phenotype in HFpEF versus a severe neurohumoral, pro-inflammatory, and often cachectic phenotype in HFrEF.

## Prognostic significance

### HFrEF

**Fig. 3:**
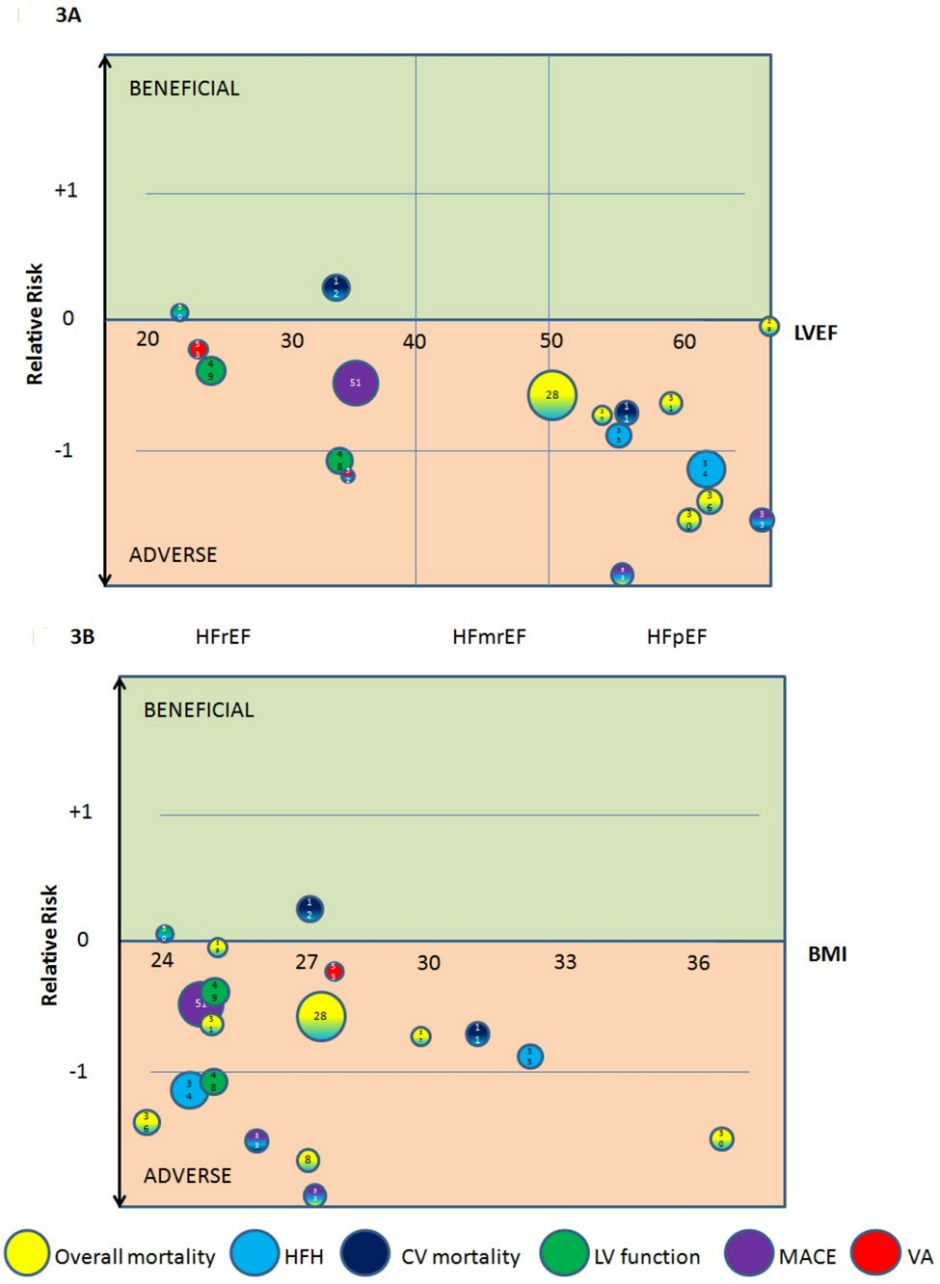
Relative risk of heart failure outcomes according to epicardial adipose tissue (EAT) amount, stratified by** A** left ventricular ejection fraction and** B **body mass index Each outcome is represented by a colored circle (see legend for color coding), with circle size proportional to the number of participants in the corresponding study (numbered references). A relative risk of − 1 indicates that the outcome occurred twice as frequently in participants with higher versus lower EAT volume or thickness. Abbreviations: CV, cardiovascular; HFH, heart failure hospitalizations; LV, left ventricle; LVEF, left ventricular ejection fraction (indicates average LVEF in a specific study); MACE, major adverse cardiovascular events; VA, ventricular arrhythmias

A total of six studies, enrolling more than 1,300 patients, investigated the association between EAT volume or thickness and outcomes in patients with HFrEF. Three studies evaluated the predictive value of EAT volume/thickness for a composite endpoint of CV deaths and HFH. Two reported that greater EAT thickness was associated with a lower risk of the composite outcome, although one demonstrated only borderline significance [21, 71]. In contrast, the third study found that increased EAT thickness predicted higher rates of both the composite endpoint and HFH [73]. Three additional studies examined whether EAT volume/thickness could predict LV recovery, a key marker of favorable prognosis. Two studies reported that more abundant EAT was associated with a reduced likelihood of LV recovery [69, 70], whereas one study observed the opposite association [73]. Finally, the sixth study demonstrated a positive association between EAT volume and MACE [72] (Fig. 3A).

### HFpEF and HFmrEF

A total of 11 studies, enrolling more than 1,500 patients, have evaluated the prognostic value of EAT in HFpEF patients. Five studies [34–38] examined the association between EAT volume or thickness and a composite endpoint of all-cause mortality and heart failure hospitalizations (HFH), while one additional study assessed a broader composite endpoint including mortality, major adverse cardiovascular events (MACE), and HFH 39 (Fig. 3). Two studies investigated cardiovascular (CV) mortality and HFH [21, 40], and further two studies focused specifically on HFH [41, 42] (Table 1).

Across all studies, more abundant EAT—assessed as volume by CT or CMR in eight studies, or as thickness by echocardiography in three studies—was consistently associated with worse clinical outcomes in HFpEF (Fig. 3). Overall, the adverse prognostic impact of more abundant EAT appeared consistent across ranges of LVEF, body mass index (BMI) (Fig. 3), sex and age, indicating that EAT reflects a disease-relevant cardiac phenotype rather than systemic obesity alone. Collectively, current evidence strongly supports EAT as a meaningful and reproducible marker of adverse prognosis in HFpEF.

However, the association with outcomes was driven primarily by HFH and follow-up durations were relatively short, ranging from 12 to 46 months. Only one study evaluated CT-derived EAT density rather than volume as a prognostic marker and demonstrated that lower EAT density predicted a composite endpoint of all-cause mortality and HFH, although questions remain regarding the independence of this association, since EAT density was inversely correlated with EAT volume in this study [43].

No specific study focused on HFmrEF patients, but they were included in two studies enrolling HFpEF patients [34,35], with results closely mimicking these obtained for the HFpEF cohort.

## Mechanisms

### EAT and ventricular arrhythmias

Since ventricular arrhythmias (VA) are major contributors to HF mortality, it is important to review studies assessing VA risk in patients with and without HF to gain insight into potential mechanisms underlying EAT effects on arrhythmogenesis, [44] in particular in view of very limited data on VA in the HFpEF cohort.

A recent study involving 98 patients with HFrEF and implanted ICDs found that EAT volume and intramyocardial fat volume, assessed by CT, were associated with VA—defined as appropriate ICD shocks or sudden arrhythmic death—in patients with non-ischemic cardiomyopathy, but not in those with ischemic cardiomyopathy [45]. LV tissue heterogeneity, reflecting the combined presence of fibrosis and intramyocardial fat, was a strong predictor of VAs. These findings underscore the importance of interactions among EAT, intramyocardial fat, and myocardial fibrosis in arrhythmogenesis.

Furthermore, patients with HFrEF and high pericardial+epicardial fat volume had more VA and increased arrhythmic mortality than those with low volume, despite similar BMI, again suggesting local rather than systemic effects of increased adiposity [46].

A Chinese study [47] demonstrated that, in a cohort of 101 patients with idiopathic VT and no structural heart disease (mean LVEF 60%), EAT volume was an independent predictor of VT recurrence. Patients with recurrent VT had approximately 50% greater EAT volume compared with those without recurrence, whereas BMI did not differ between the groups.

Another retrospective Chinese study [48] found that EAT volume was an independent predictor of idiopathic VT in patients without structural heart disease; moreover, patients with high EAT volume had longer Tp-e intervals and increased Tp-e/QTc ratios on electrocardiogram, suggesting greater dispersion of repolarization.

In a separate study of 61 patients, EAT volume was identified as an independent predictor of VT recurrence following successful catheter ablation [49]. Most patients with recurrent VT had HFrEF (mean LVEF 29%), and 80% had an implanted cardioverter-defibrillator (ICD). Notably, EAT volume was unrelated to BMI, and EAT thickness measured over the right atrioventricular groove demonstrated the highest predictive value.

Overall, all studies indicate that increased EAT volume is independently associated with a higher incidence of VA. However, the limited available data do not allow precise identification of the underlying arrhythmogenic mechanisms.

### EAT, obesity and HF outcomes

Analyses demonstrate that EAT volume or thickness is an independent predictor of HF outcomes, in particular unrelated to measures of systemic obesity. This conclusion is further supported by analyses across different BMI categories within individual clinical studies (Fig. 3B), which show that the association between EAT and heart failure outcomes is preserved across BMI ranges, with no modifying effect of BMI.

### Physiological mechanisms

HFpEF is characterized not only by higher EAT volume and thickness versus healthy subjects, but also high EAT abundance correlates with impairment of LV diastolic and systolic function [33], RV function [50], increased RV mass [51] and impaired exercise capacity as well as with increased LV and RV filling pressures [32] and, as shown above, with and worse clinical prognosis. While such correlations do not necessitate causal relation, EAT appears to act as an active contributor to disease progression rather than a passive bystander.

EAT can contribute to HFpEF through mechanical, metabolic, and paracrine mechanisms. Fatty infiltration from EAT into the adjacent myocardium can disrupt myocardial ultrastructure and alter electrophysiological properties, ultimately promoting cardiac hypertrophy and diastolic dysfunction [52]. In addition, expansion of EAT within the pericardial space increases pericardial constraint, resulting in cardiac compression and elevated left and right ventricular filling pressures, particularly during exercise [53]. This contributes to pulmonary congestion, interventricular septal shift, impaired left ventricular filling, and reduced exercise capacity, effects that are especially evident in HFpEF patients with high EAT volume [54]. Metabolically, excess circulating free fatty acids, typically found in heart failure, promote myocardial lipid accumulation, which can result in lipotoxicity, mitochondrial dysfunction, and impaired cardiomyocyte function [55]. In HFpEF, triglyceride accumulation predominates, whereas in HFrEF impaired fatty acid oxidation and reduced DGAT activity favor accumulation of more toxic lipid intermediates such as diacylglycerol. EAT may also exert a buffering role against lipid overload, although this remains unproven and may be particularly relevant in HFrEF [56]. Additionally, EAT in obesity and HFpEF adopts a proinflammatory and profibrotic phenotype, secreting mediators such as activin A that impair cardiomyocyte calcium handling and contractile function [57].

The relationship between HFrEF and EAT is more nuanced, complex and less understood. First, while the total amount of EAT is usually increased, the myocardial coverage or EAT thickness are usually reduced. Second, low EAT amount correlates with impaired LV systolic [22–25] and diastolic [29, 58] function, LV fibrosis [24] and impaired exercise capacity [21] in HFrEF. Third, as outlined above, prognostic data in HFrEF remain inconsistent. Some studies suggest that greater EAT thickness or volume is associated with more favorable outcomes or a higher likelihood of LV reverse remodeling, whereas others link increased EAT to worse clinical outcomes or heightened arrhythmic risk. These discrepancies likely reflect competing biological mechanisms, whereby moderate amounts of EAT may exert metabolically protective effects, while excessive or pathologically remodeled EAT may promote inflammation, fibrosis, and arrhythmogenesis.

Our data indicate that EAT exhibits substantial heterogeneity in both volume and density, with density potentially reflecting the extent of inflammatory remodeling within EAT [16, 59]. This field therefore requires further investigation, focusing not only on underlying mechanisms but also on better parameters of EAT remodeling, such as epicardial adipose tissue density [20]. Ultimately, these findings suggest that an individualized approach to EAT assessment in HFrEF may be necessary.

Although data in HFmrEF remain limited, available evidence indicates that the prognostic role of EAT in HFmrEF closely resembles that observed in HFpEF. This supports the concept that HFmrEF—at least in a substantial subset of patients—shares EAT-related pathophysiological mechanisms with HFpEF.

The differential role of EAT across heart failure subtypes is further highlighted by the effects of various therapies. Sodium–glucose cotransporter 2 (SGLT2) inhibitors reduce EAT volume and are associated with improved outcomes in both HFrEF and HFpEF, with EAT reduction correlating with remission of diastolic dysfunction in selected populations [60]. Glucagon-like peptide 1 (GLP-1) receptor agonists also reduce EAT [61]; however, clinical trials suggest a lack of benefit—and even a potential signal of harm—in HFrEF [62, 63], while demonstrating beneficial effects in HFpEF [64]. Bariatric surgery leads to substantial and sustained reductions in EAT volume [65], accompanied by improvements in left ventricular diastolic function [66] and a lower risk of heart failure compared with individuals with obesity who do not undergo surgical treatment [67]. Collectively, these findings indicate that EAT reduction alone does not uniformly translate into clinical benefit in HF. Future studies are needed to determine whether targeted modification of EAT may serve as a therapeutic strategy or as a marker of treatment response in specific HF phenotypes.

## Conclusions

EAT emerges as a promising, imaging-derived biomarker for risk stratification in HFpEF and HFmrEF, with potential utility in identifying patients at higher risk for recurrent hospitalizations and adverse outcomes. In HFrEF, future studies should focus on EAT quality (density, inflammatory profile), spatial distribution, and its interaction with fibrosis and intramyocardial fat rather than volume alone. Prospective studies are required to determine whether EAT is a modifiable therapeutic target and whether interventions that reduce or normalize EAT can translate into improved clinical outcomes.

## Data Availability

No datasets were generated or analysed during the current study.
